# Silencing Mist1 Gene Expression Is Essential for Recovery from Acute Pancreatitis

**DOI:** 10.1371/journal.pone.0145724

**Published:** 2015-12-30

**Authors:** Anju Karki, Sean E. Humphrey, Rebecca E. Steele, David A. Hess, Elizabeth J. Taparowsky, Stephen F. Konieczny

**Affiliations:** Department of Biological Sciences, the Purdue Center for Cancer Research, and the Bindley Bioscience Center, Purdue University, West Lafayette, IN, 47907–2057, United States of America; Centro Nacional de Investigaciones Oncológicas (CNIO), SPAIN

## Abstract

Acinar cells of the exocrine pancreas are tasked with synthesizing, packaging and secreting vast quantities of pro-digestive enzymes to maintain proper metabolic homeostasis for the organism. Because the synthesis of high levels of hydrolases is potentially dangerous, the pancreas is prone to acute pancreatitis (AP), a disease that targets acinar cells, leading to acinar-ductal metaplasia (ADM), inflammation and fibrosis—events that can transition into the earliest stages of pancreatic ductal adenocarcinoma. Despite a wealth of information concerning the broad phenotype associated with pancreatitis, little is understood regarding specific transcriptional regulatory networks that are susceptible to AP and the role these networks play in acinar cell and exocrine pancreas responses. In this study, we examined the importance of the acinar-specific maturation transcription factor MIST1 to AP damage and organ recovery. Analysis of wild-type and *Mist1* conditional null mice revealed that *Mist1* gene transcription and protein accumulation were dramatically reduced as acinar cells underwent ADM alterations during AP episodes. To test if loss of MIST1 function was primarily responsible for the damaged status of the organ, mice harboring a Cre-inducible *Mist1* transgene (*iMist1*) were utilized to determine if sustained MIST1 activity could alleviate AP damage responses. Unexpectedly, constitutive *iMist1* expression during AP led to a dramatic increase in organ damage followed by acinar cell death. We conclude that the transient silencing of *Mist1* expression is critical for acinar cells to survive an AP episode, providing cells an opportunity to suppress their secretory function and regenerate damaged cells. The importance of MIST1 to these events suggests that modulating key pancreas transcription networks could ease clinical symptoms in patients diagnosed with pancreatitis and pancreatic cancer.

## Introduction

The majority of the exocrine pancreas consists of acinar cells which are tasked with synthesizing, modifying, packaging and secreting vast quantities of pro-digestive enzymes (zymogens) into the duodenum to maintain metabolic homeostasis for the organism [1–4]. The ability of acinar cells to produce high levels of appropriately packaged proteins requires the coordination of pathways responsible for the accumulation and assembly of critical components of the secretory apparatus, the establishment of proper apical-basal polarity and cell-cell communication and the proper management of mis-folded proteins through the Unfolded Protein Response (UPR) [3, 5–9]. Because of the high levels of potentially dangerous hydrolases synthesized by the exocrine pancreas, the organ is prone to a number of disease states including pancreatitis and pancreatic cancer.

Pancreatitis is a disease that targets pancreatic acinar cells, leading to organ inflammation, fibrosis and overall tissue disruption [10]. It is commonly associated with gallstones and excessive alcohol consumption which leads to cell damage through intracellular activation of zymogens [11]. Importantly, pancreatitis is also a known risk factor for pancreatic ductal adenocarcinoma (PDAC) [12–14] and a number of mouse genetic studies have shown that episodes of acute pancreatitis (AP) can serve as a driving force for KRAS^G12D^-induced PDAC [15–23]. Indeed, a hallmark of AP is alteration of acinar cell identity where acinar cells acquire ductal characteristics through a process known as acinar-ductal metaplasia (ADM) [20, 21, 24, 25]. ADM is thought to represent a precursor state that can progress to PDAC under conditions of oncogenic and tumor suppressor mutations [16–18, 26–30]. Despite a wealth of information concerning the broad phenotype associated with pancreatitis, little is understood regarding the transcriptional regulatory networks that are susceptible to AP episodes and how these networks allow acinar cells and the exocrine organ to recover.

Key transcription factors that establish and maintain a healthy acinar cell state include PTF1A, MIST1 (also known as BHLHA15), GATA6, and NR5A2 [3, 31–38]. PTF1A and MIST1 are basic helix-loop-helix (bHLH) factors that have been shown to exhibit tumor suppressor properties where acinar cells lacking each factor are highly susceptible to KRAS^G12D^-induced transformation [26, 39, 40]. Both factors play important roles in acinar differentiation events. PTF1A is essential for *Mist1* gene expression and expression of most zymogen encoding genes including *Elastase*, *Carboxypeptidase* and *Amylase* [32, 41–43]. Although not essential for embryonic acinar development, MIST1 plays an essential role in the maturation of acinar cells by regulating genes critical for apical-basal cell polarity, the assembly and clustering of secretory granules, proper Ca^2+^ signaling, the expansion of the endoplasmic reticulum (ER), UPR pathway homeostasis, cell cycle progression and regulated exocytosis [33, 44–50]. What sets MIST1 apart from PTF1A is that it exhibits a broad tissue specificity, being present in most serous secretory cells in the body, including salivary acinar, stomach zymogenic, mammary alveolar and immunoglobulin secreting B cells [51–57]. In all cases, MIST1 is responsible for the overall upregulation of the protein synthesis, processing and secretory machinery, often acting as a scaling factor to insure highly efficient regulated secretion for each cell type [31, 45, 52].

The importance of MIST1 to maintaining a healthy cellular state for secretory cells is also evident in a number of different cancers. Both stomach cancer and PDAC tumors have been shown to initiate from *Mist1*-expressing secretory cells [26, 27, 58–60]. However, early in the transformation process, stomach zymogenic cells and pancreatic acinar cells that are undergoing metaplasia silence *Mist1* gene expression, suggesting that inhibiting MIST1 activity is a critical step in allowing cells to enter into a proliferative phase [26, 39, 46, 59–61]. Furthermore, sustained *Mist1* expression in *Kras* ^*G12D*^-expressing acinar cells inhibits ADM and PDAC development, again highlighting the concept that MIST1 exhibits tumor suppressor properties [26, 39].

Because pancreatitis is a known risk factor for PDAC, and MIST1 is critical to PDAC development, we set out to examine if *Mist1* gene expression is silenced under AP conditions and to test if sustained MIST1 activity would alleviate AP damage responses. Our studies demonstrate that during AP damage in both mouse and human, *Mist1* gene transcription and protein accumulation are dramatically reduced. In mice subjected to caerulein-induced AP, *Mist1* silencing is a transient event. As cells recover from AP damage, the *Mist1* locus is transcriptionally re-activated and MIST1 protein levels are restored. Despite this re-expression, analysis of conditional *Mist1* knock-out (*Mist1 cKO*) mice revealed that *Mist1*-deficient pancreata responded similarly to AP treatment as control animals, with an initial damage phase that was rapidly followed by recovery. We next examined if sustained *Mist1* expression (*iMist1*) in genetically engineered mice could alleviate AP-induced damage. Surprisingly, in *iMist1* animals, AP produced a dramatic phenotype of significant tissue damage followed by cell death in cells that expressed *iMist1*. Despite the extreme damaged response in *iMist1* pancreata, the pancreas partially recovered by regenerating healthy acini from the small minority of acinar cells that failed to activate the *iMist1* transgene. We conclude that silencing *Mist1* expression is a critical event for acinar cells to survive an AP episode where down regulating MIST1 activity may allow cells to suppress their secretory function and permit a window of cell proliferation. However, to fully re-establish a functional acinar cell capable of efficient exocytosis, the *Mist1* gene must be reactivated to scale up the appropriate intracellular machinery that generates secretory vesicles, expands the ER and establishes cell communication via gap junction signaling. The importance of MIST1 to these events suggests that devising strategies to modulate transcriptional networks could ease clinical symptoms in patients diagnosed with pancreatitis and pancreatic cancer.

## Materials and Methods

### Mouse Strains and Genotyping

*Mist1* ^*CreERT/+*^ and *LSL-Mist1* ^*myc*^ (*iMist1* ^*myc*^) mice have been described previously [26, 33, 58]. *Mist1* ^*lox/+*^ mice were produced by generating a *Mist1* targeting vector containing *loxP* sites flanking the entire *Mist1* coding region within exon 2 [62]. In addition, a small biotin-tag [63] and MYC-tag were added to the N-terminus and C-terminus of the MIST1 open reading frame, respectively. ES cell electroporation and blastocyst injections were performed by the Purdue University Transgenic Mouse Core Facility. *Mist1* conditional knock-out (*Mist1 cKO*) mice (*Mist1* ^*CreERT/lox*^) were produced by crossing *Mist1* ^*CreERT/+*^ mice to *Mist1* ^*lox/+*^ animals. Induction of CreER^T2^ activity was accomplished by administrating tamoxifen (200 μl of 20 mg/ml) via oral gavage to adult mice (6–8 wk). Genotyping primer sets are listed in S1 Table. All experiments were performed with mice on a C57BL/6 background and all animal studies were conducted in strict compliance with the recommendations in the Guide for the Care and Use of Laboratory Animals of the National Institutes of Health and the Purdue University IACUC guidelines. The protocol was approved by the IACUC Committee of Purdue University (Approval Number 1110000037).

### Acute Pancreatitis Induction

AP was induced by caerulein via intraperitoneal (*i*.*p*) injections. Adult mice (6–8 wk) were given eight hourly *i*.*p*. injections of caerulein (Sigma-Aldrich, St. Louis, MO) for two consecutive days (50 μg/kg body weight). Control mice received PBS. Mice were sacrificed and pancreata samples were harvested for paraffin blocks, protein and RNA at various times (6h → 8w) following the last caerulein injection (set to 0h). In some instances, mice were given BrdU (5-bromo-2'-deoxyuridine) (200 μl of 10 mg/ml) by *i*.*p*. six hr prior to sacrificing. For all analyses, 3–7 mice per time point/genotype/experimental condition were analyzed.

### Histology and Immunohistochemistry

Mouse pancreata were fixed in 10% neutral buffered formalin, embedded in paraffin, sectioned and stained using conventional histological techniques. Tissue sections (5 μm) were deparaffinized and retrieved using the 2100-Retriever (Electron Microscopy Sciences, Hatfield, PA) with antigen unmasking solution (Vector Laboratories, Burlingame, CA). For IHC, sections were incubated in 3% H_2_O_2_ for 5 min to block endogenous peroxidase activity followed by 1 hr in M.O.M. blocking reagent (Vector Laboratories, Burlingame, CA). Tissue sections were incubated in primary antibodies for 1 hr at room temperature. Biotinylated secondary antibodies were used at 1:200 dilution for 20 min at room temperature. IHC development was performed using Vector reagents and DAB (diamonibenzidine) peroxidase substrate (Vector Labs, Burlingame, CA). Secondary antibodies for immunofluorescence utilized avidin-conjugated Alexa Fluor 488, Alexa Fluor 594, Alexa Fluor 555, Oregon Green 488, and Alexa Avidin Cy5.5 (Invitrogen, Camarillo, CA). Detailed information on the primary antibodies used in this study is provided in S2 Table

### Immunoblots

Pancreata samples were lysed using a Tissue Tearor Homogenizer (Biospec Products, Inc) in ice-cold RIPA buffer supplemented with protease inhibitors, phosphatase inhibitors and sodium orthovanadate. Protein extracts (30 μg) were resolved on 12% SDS-PAGE and transferred onto PVDF membranes (Bio Rad, Hercules, CA). Membranes were blocked overnight at 4°C in 5% non-fat dry milk prepared in Tris-buffered saline plus 0.1% Tween 20. Membranes were incubated in primary antibodies at room temperature for 1 hr followed by three 10 min washes and then incubated in horseradish peroxidase (HRP) conjugated secondary antibodies at 1:5000 dilution at room temperature for 30 min. Immunoblots were visualized on X-ray films using an enhanced chemiluminescence (ECL) kit (Thermo Scientific, Waltham, MA) and quantified using ImageJ (normalized to the HSP90 or S6 signal) or visualized and quantified on a ChemiDoc Touch Imaging System (Bio Rad, Hercules, CA) using the HSP90 or S6 signal for normalization.

### RNA Expression Analysis

Total cellular RNA from pancreata was isolated using the E.Z.N.A midi kit (Qiagen, Valencia, CA). For quantitative RT-PCR analysis, reverse transcription using 1 μg RNA was performed with the iScript cDNA synthesis kit (Bio-Rad, Hercules, CA), followed by gene amplification using FastStart Universal SYBR Green (Roche Applied Science, Indianapolis, IN) and a Roche LightCycler 96 thermocycler (Roche Diagnostics Corporation, Indianapolis, IN). All individual reactions were performed in duplicate and all genes were normalized to *18S* ribosomal RNA or to the ribosomal transcript *Rplp0*. Quantitative RT-PCR primers are listed in S3 Table. GraphPad Prism 6 (La Jolla, CA) was used to generate graphs included in this study. Statistical analyses are presented as standard error of the mean. P values were determined using two-tailed unpaired tests.

### Microscopy and Image Analysis

All H&E, IHC and IF images were taken using an Olympus BX51 upright microscope and a DP80 high resolution camera (Olympus Life Science). Images representing INSULIN+, AMYLASE+, MYC+, MYC-, BrdU+, BrdU-, etc. areas were quantified using ImageJ (NIH) from 12–15 random 10x fields from ≥ 3 sections at different pancreas depths per mouse. For each image, individual pixels were converted to μm to establish the appropriate area in μm^2^. All calculations were performed in GraphPad Prism 6. Statistical analyses are presented using standard error of the mean. P values were determined using two-tailed unpaired tests.

## Results

### Mist1 Gene Expression Is Transiently Silenced upon Acute Pancreatitis Damage

The MIST1 transcription factor (also known as BHLHA15) regulates key genes that are required for acinus polarity, cell-cell junctions and the processing of zymogen granules [31, 33, 44, 56]. Loss of MIST1 function leads to deficiencies in acinar cell integrity, cell polarity, ER expansion and regulated exocytosis [33, 48, 50, 64, 65]. Similar defects in polarity and acinar cell properties are also hallmarks of *Kras* ^*G12D*^-driven transformation events where acinar cells exhibit acinar-ductal metaplasia (ADM) that progresses to pancreatic intraepithelial neoplasia (PanIN) and pancreatic ductal adenocarcinoma (PDAC) [26, 39, 58]. PanINs and PDAC tumors, each derived from acinar cells, lose acinar characteristics and no longer express MIST1 protein [2, 26, 27, 39, 66]. The importance of cell integrity to PDAC disease is also supported by studies showing that damage to *Kras* ^*G12D*^-expressing acinar cells via an episode of acute pancreatitis (AP) accelerates PanIN formation [15, 18, 19, 21]. In all cases, acinar-derived PanIN/PDAC epithelial cells remain MIST1 negative.

**Fig 1:**
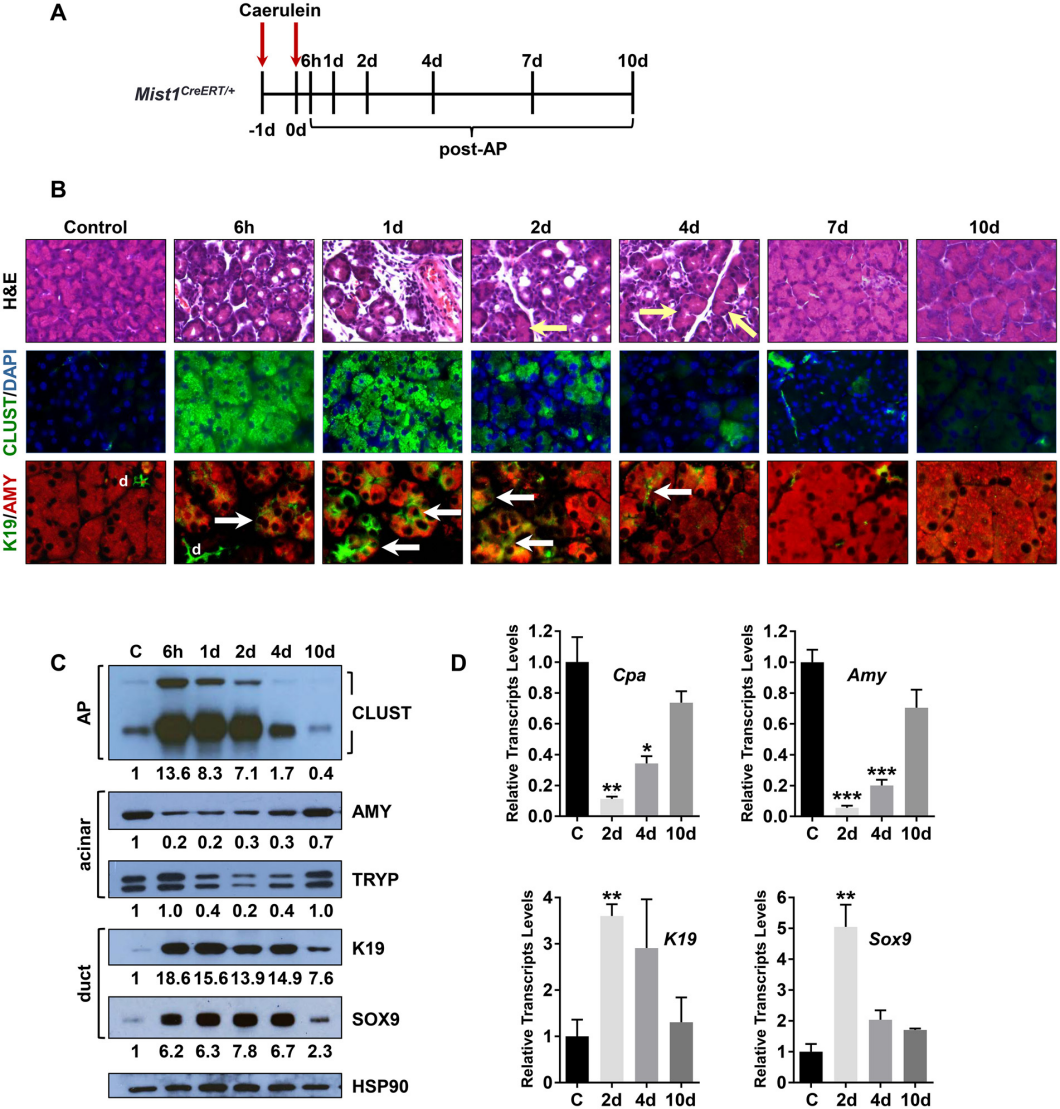
Characterization of Mist1^CreERT/+^ mice following acute pancreatitis. (A) Time course diagram of caerulein-induced acute pancreatitis. (B) H&E and IF analyses of *Mist1* ^*CreERT/+*^ pancreas samples in the absence of AP treatment (control) or post-AP for the indicated times. At early times acinar cells exhibit elevated levels of Clusterin expression and Amylase+/K19+ ADM lesions. However, by 10d post-AP the majority of the tissue fully recovers. Arrows in the H&E section point out recovered acini whereas arrows in the K19/AMY panels indicate ADM lesions. (d, duct) (C) Immunoblot analysis of *Mist1* ^*CreERT/+*^ pancreata post-AP. HSP90 was used as a loading control. Relative expression levels are indicated below each panel, normalized to the corresponding HSP90 signal. (D) RT-qPCR analysis of gene transcripts confirms the initial ADM phenotype followed by recovery at 10d post-AP. *p ≤ 0.05; **p ≤ 0.01; ***p ≤ 0.001.

To evaluate the importance of MIST1 during acinar metaplasia, we characterized *Mist1* expression during the damage and subsequent recovery phases of AP, a known driver of PDAC tumor development [15, 18, 19, 21]. For these studies, *Mist1* ^*CreERT/+*^ mice were used as controls as all subsequent mouse lines contained the *Mist1* ^*CreERT*^ knock-in allele [26, 58]. Standard caerulein treatment (Fig 1A) of 8 week *Mist1* ^*CreERT/+*^ mice led to significant and rapid damage to the exocrine acinar cells. As early as 6h post-AP, acinar lumens were distended and zymogen granules were rapidly lost (Fig 1B and S1A Fig). By 1d post-AP, significant increases in edema and inflammatory cell infiltrates were observed, accompanied by extensive formation of KERATIN19 (K19)+/AMYLASE (AMY)+ ADM lesions. Expression of CLUSTERIN, a known marker of acinar cell damage [67, 68], also was greatly elevated at 6h post-AP (Fig 1B,1C and S1A,S1B Fig). Transcript and protein levels of acinar cell markers, including *Amylase* (*Amy*), *Trypsinogen* (*Tryp*) and *Carboxypeptidase* (*Cpa*), were significantly reduced over the 6h-2d post-AP period (Fig 1C,1D and S1B Fig). In contrast, ductal markers (K19, SOX9) were greatly elevated, confirming the formation of extensive ADM (Fig 1B–1D, S1B Fig). Identical ADM responses were obtained with caerulein-treated wild-type mice (data not shown). Despite significant development of ADM lesions upon AP induction, AP metaplasia was transient as lesions resolved 4d-10d post-AP. In all cases, *Clusterin*, *K19* and *Sox9* transcript and protein levels returned to their low control states while acinar markers (*Amylase*, *Trypsinogen*, *Carboxypeptidase*) re-established high expression thresholds (Fig 1B–1D and S1A,S1B Fig).

**Fig 2:**
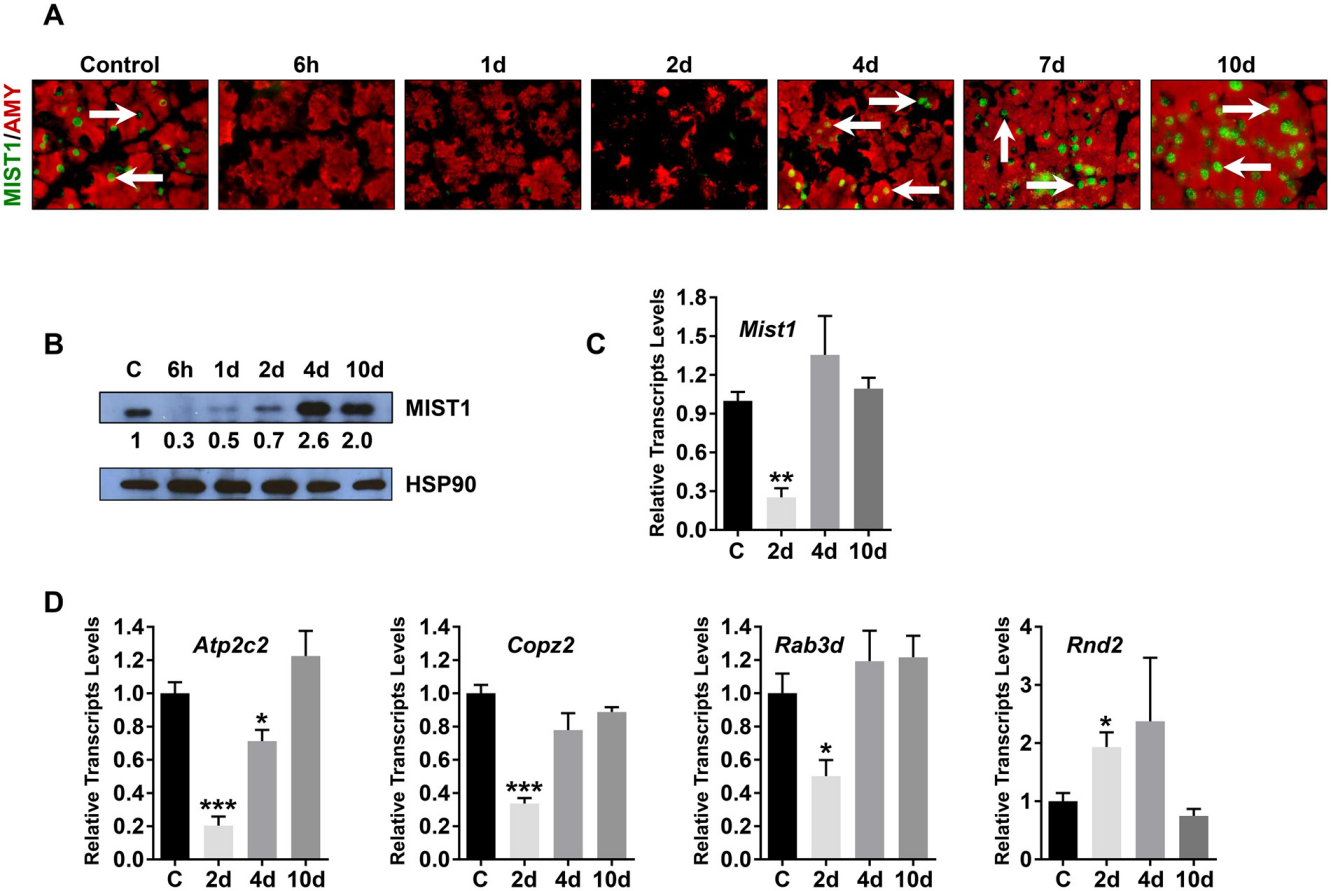
The *Mist1* gene is transcriptionally silenced during acute pancreatitis. (A) Analysis of MIST1 (arrows) in *Mist1* ^*CreERT/+*^ pancreata. Damaged acinar cells exhibit greatly reduced MIST1 levels that recover by 10d post-AP. (B) MIST1 immunoblot analysis of *Mist1* ^*CreERT/+*^ pancreata over the indicated post-AP time points. HSP90 was used as a loading control. Relative expression levels are indicated below the MIST1 panel, normalized to the corresponding HSP90 signal. (C) RT-qPCR analysis of *Mist1* gene expression during AP damage and recovery. (D) RT-qPCR of MIST1 gene targets during AP. *p ≤ 0.05; **p ≤ 0.01; ***p ≤ 0.001.

The major phenotype associated with caerulein-induced AP is loss of acinar cell integrity [22, 69–71]. Because the transcription factor MIST1 is critical for maintaining acinar cell polarity and function, we examined if MIST1 protein accumulation was altered in AP mice. As shown in Fig 2A and 2B, high levels of MIST1 protein were detected in all control acinar cells, whereas duct and islet cells remained MIST1 negative (S2 Fig). However, in AP mice, *Mist1* transcripts and protein were rapidly lost in damaged acinar cells (Fig 2A–2C). The absence of MIST1 was observed 6h-2d post-AP during the period corresponding to the major time frame for ADM lesion induction. Nonetheless, as mouse acinar cells recovered (4d-10d post-AP), *Mist1* transcript and protein levels greatly increased, achieving levels that were comparable to those observed in control acinar cells. The transient change in *Mist1* transcripts and protein during the AP response was also reflected in the expression profiles of known MIST1 target genes [33, 44, 56]. Transcripts from MIST1-induced genes *Atp2c2*, *Copz2* and *Rab3d* were reduced during the 6h-2d post-AP period while transcripts from MIST1-repressed genes (*e*.*g*., *Rnd2*) were up-regulated (Fig 2D). Thus, transient silencing of *Mist1* influences a number of key events associated with acinar cell integrity. These results suggest that the process of silencing and then re-expressing *Mist1* may be critical in allowing the exocrine pancreas to properly recover from an acute pancreatitis episode.

### Generation and Characterization of Mist1^lox/lox^ Mice

**Fig 3:**
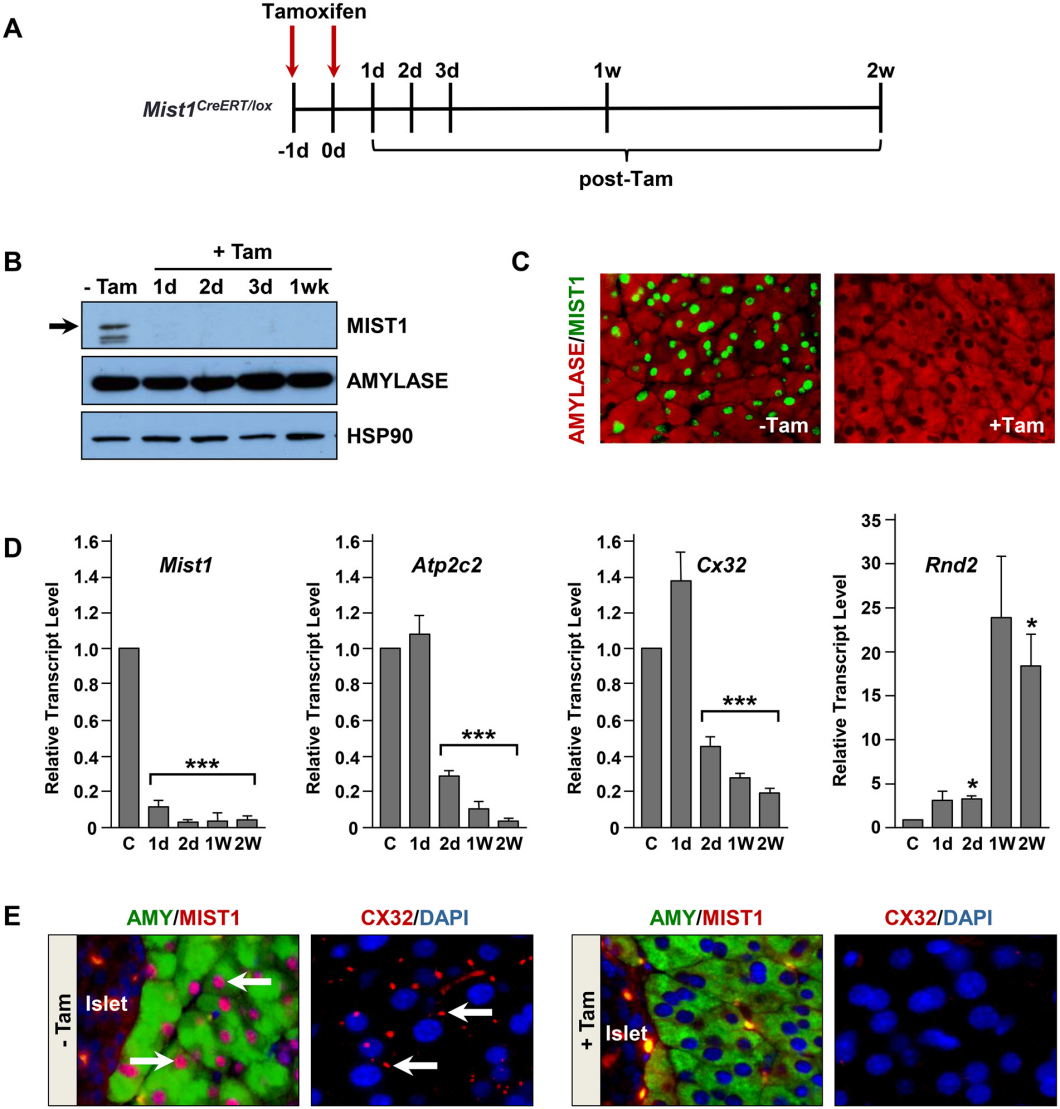
Establishing the Mist1^CreERT/lox^ model system. (A) Schematic of Tam treatment and time course analysis for *Mist1* ^*CreERT/lox*^ mice. (B) Immunoblot demonstrating the absence of MIST1 protein in pancreata from Tam-treated *Mist1* ^*CreER/lox*^ mice. HSP90 was used as a loading control. (C) IF staining with anti-MIST1 confirming that the vast majority of acinar cells are MIST1 negative 7d post-Tam treatment. (D) RT-qPCR analysis of MIST1 gene targets revealing loss of MIST1 regulation post-Tam. (E) CX32 gap junctions are readily detected in pancreata from -Tam treated *Mist1* ^*CreERT/lox*^ mice but are completely absent in +Tam samples. *p ≤ 0.05; ***p ≤ 0.001.

Previous studies reported that *Mist1* null animals exhibited a pronounced AP phenotype, suggesting that the absence of MIST1 sensitizes acinar cells to an AP episode [70, 72, 73]. However, because these studies could only use germ line *Mist1* nulls, it was not possible to establish if the enhanced AP phenotype was due to embryonic loss of MIST1 protein or was the result of inducing AP in already damaged adult pancreata. Thus, to directly test if MIST1 protein is required for acute pancreatitis recovery, we generated and characterized a conditional *Mist1* ^*lox/lox*^ mouse line (S3 Fig). *Mist1* ^*CreERT/+*^ mice were crossed to *Mist1* ^*lox/+*^ animals to generate *Mist1* ^*CreERT/lox*^ offspring where one *Mist1* allele expressed CreER^T2^ while the other *Mist1* allele, engineered with an N-terminal BT-tag and a C-terminal MYC-tag, was flanked by LoxP sites (S3 Fig). Treatment of 8 wk *Mist1* ^*CreERT/lox*^ mice with tamoxifen (Tam) led to efficient recombination and rapid loss of MIST1 protein in 99.6% acinar cells as early as 24h post-Tam (Fig 3A–3C). Deletion of *Mist1* also led to significant changes in the expression patterns of MIST1 target genes. As predicted, expression of *Atp2c2* and *Cx32* decreased while *Rnd2* gene transcripts (which are normally repressed by MIST1 protein) increased following Tam treatment (Fig 3D). Similarly, MIST1-regulated CX32 gap junctions [33, 49] were rapidly lost upon Tam treatment of *Mist1* ^*CreERT/lox*^ mice (Fig 3E).

### Mist1^CreERT/lox^ Mice Exhibit Similar AP recovery as Mist1^CreERT/+^ Animals

**Fig 4:**
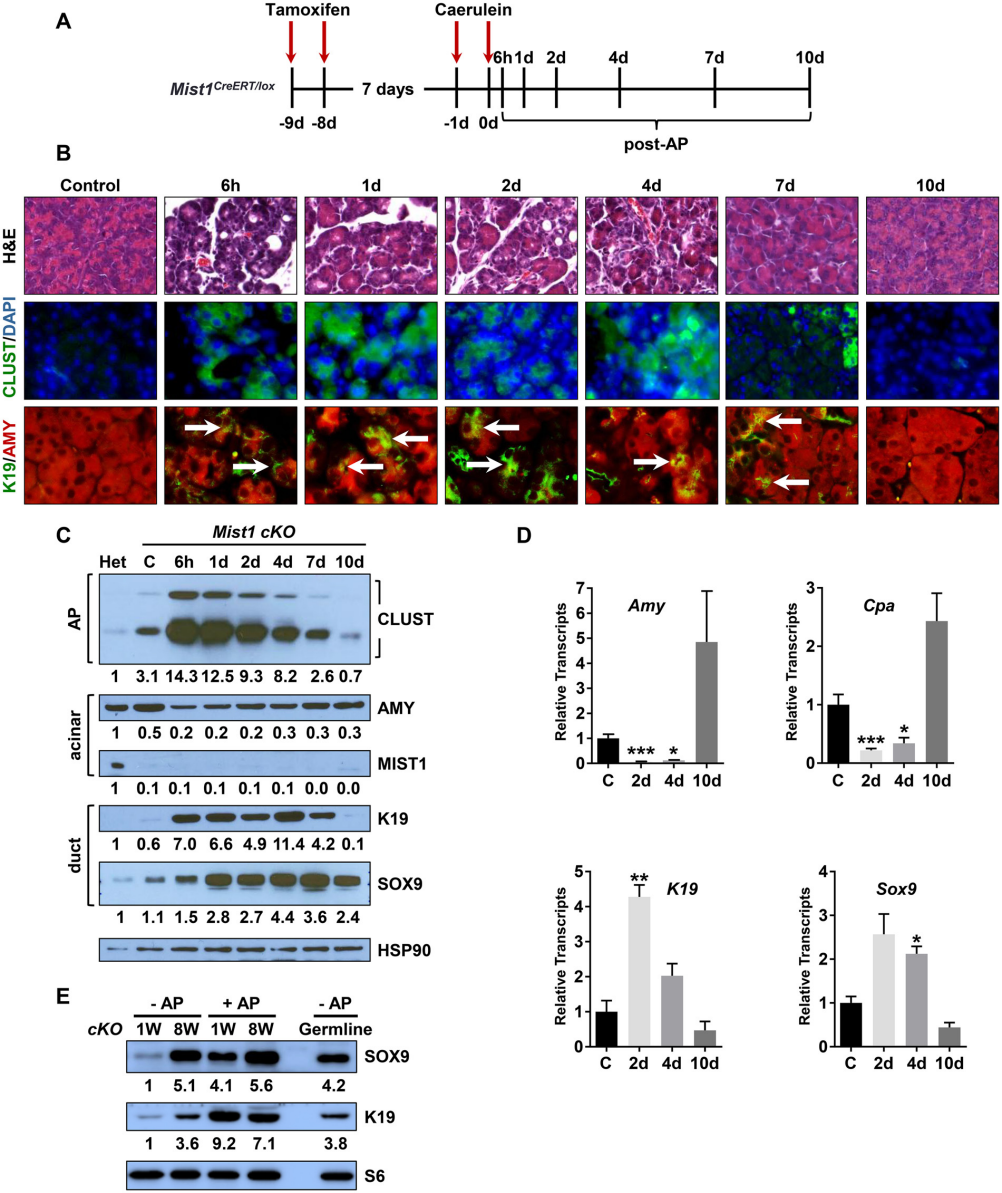
Characterization of Mist1^CreERT/lox^ mice following acute pancreatitis. (A) Time course diagram of caerulein-induced acute pancreatitis in *Mist1* ^*CreERT/lox*^ mice (*Mist1 cKO*). (B) H&E and IF analyses of *Mist1 cKO* pancreas samples in the absence of AP treatment (control) or post-AP for the indicated times. Arrows indicate AMY+/K19+ ADM lesions. (C) Immunoblot analysis of protein expression from *Mist1 cKO* samples post-AP. Het is a control *Mist1* ^*CreERT/+*^ sample. HSP90 was used as a loading control. Relative expression levels are indicated below each panel, normalized to the corresponding HSP90 signal. (D) RT-qPCR analysis of acinar and duct gene products during an AP time course study. (E) Comparison of 1 week and 8 week post-*Mist1* deletion in the *Mist1 cKO* model. *Mist1* ^*CreERT/lox*^ mice were treated with Tam and then analyzed for protein expression at the indicated times for -AP and +AP groups. *Mist1* ^*CreERT/CreERT*^ (germline *Mist1* null) mice were used as a reference control. Protein S6 was used as a loading control. Relative expression levels are indicated below each panel, normalized to the corresponding S6 signal. *p ≤ 0.05; **p ≤ 0.01; ***p ≤ 0.001.

To determine if AP-induction in *Mist1* ^*CreERT/lox*^ animals produced a recovery delay when compared to *Mist1* ^*CreERT/+*^ mice, *Mist1* ^*CreERT/lox*^ animals were treated with Tam (to delete the *Mist1* coding region) (*Mist1 cKO*) and then induced with caerulein to generate an AP response (Fig 4A). As expected, control and AP-treated *Mist1 cKO* mice failed to express MIST1 protein (Fig 4C). Caerulein injections in *Mist1 cKO* animals elicited strong edema, inflammatory cell infiltrates and extensive ADM lesions as early as 6h post-AP (Fig 4B and S4A Fig). ADM was accompanied by significant increases in *Clusterin*, *K19* and *Sox9* transcript and protein levels with a concomitant decrease in *Amylase* and *Carboxypeptidase* levels (Fig 4B–4D and S4A,S4B Fig). As with *Mist1* ^*CreERT/+*^ mice, the ADM phenotype was transient and the *Mist1 cKO* pancreas returned to a relatively normal status by 10d post-AP, although *Mist1 cKO* acini remained defective in acinar cell polarity and organization due to the absence of MIST1 protein. Surprisingly, with the exception of sustained elevated SOX9 protein levels at 10d post-AP, there was little difference between the AP responses for *Mist1* ^*CreERT/+*^ (Fig 1) and *Mist1 cKO* animals (Fig 4).

The ability of *Mist1 cKO* pancreata to recover from an acute pancreatitis episode with the same kinetics as *Mist1* ^*CreERT/+*^ mice was surprising given previous reports showing that *Mist1* null pancreata exhibited an enhanced AP response [70, 72, 73]. The main difference between the two models is that with germline *Mist1* ^*-/-*^ mice, the pancreas is significantly disorganized and defective by 8 wk of age [48]. In contrast, *Mist1* ^*CreERT/lox*^ mice allow us to delete the *Mist1* allele in adult animals and induce AP prior to the development of overt pancreas damage caused by the absence of MIST1. Thus, to establish if short versus long-term loss of MIST1 activity differentially influences AP responses, *Mist1* ^*CreERT/lox*^ animals were given Tam and then treated with caerulein at 1 week post-Tam or 8 week post-Tam. As shown in Fig 4E, even in the absence of AP, *Mist1 cKO* pancreata at 8 week post-Tam exhibited early signs of ADM, with large increases in SOX9 and K19 protein levels (compare -AP 1 week versus 8 week). The increase in ductal gene expression reflected the ADM damage response that was observed in adult *Mist1* ^*CreERT/CreERT*^ (*Mist1* null) animals where the *Mist1* locus was absent in the germline. Interestingly, AP episodes in 1 week versus 8 week post-*Mist1* deletion did not reveal a significant difference in how the pancreas responded to this acute damage (Fig 4E). In all cases, 1 week and 8 week post-tam treated mice still managed to recover from the bulk of AP-induced damage by 10d post-AP (data not shown). Taken together, we conclude that the absence of MIST1 protein in adult acinar cells has little impact in allowing cells to recover from acute pancreatitis.

### Preventing Mist1 Gene Silencing Significantly Alters the Acinar AP Response

**Fig 5:**
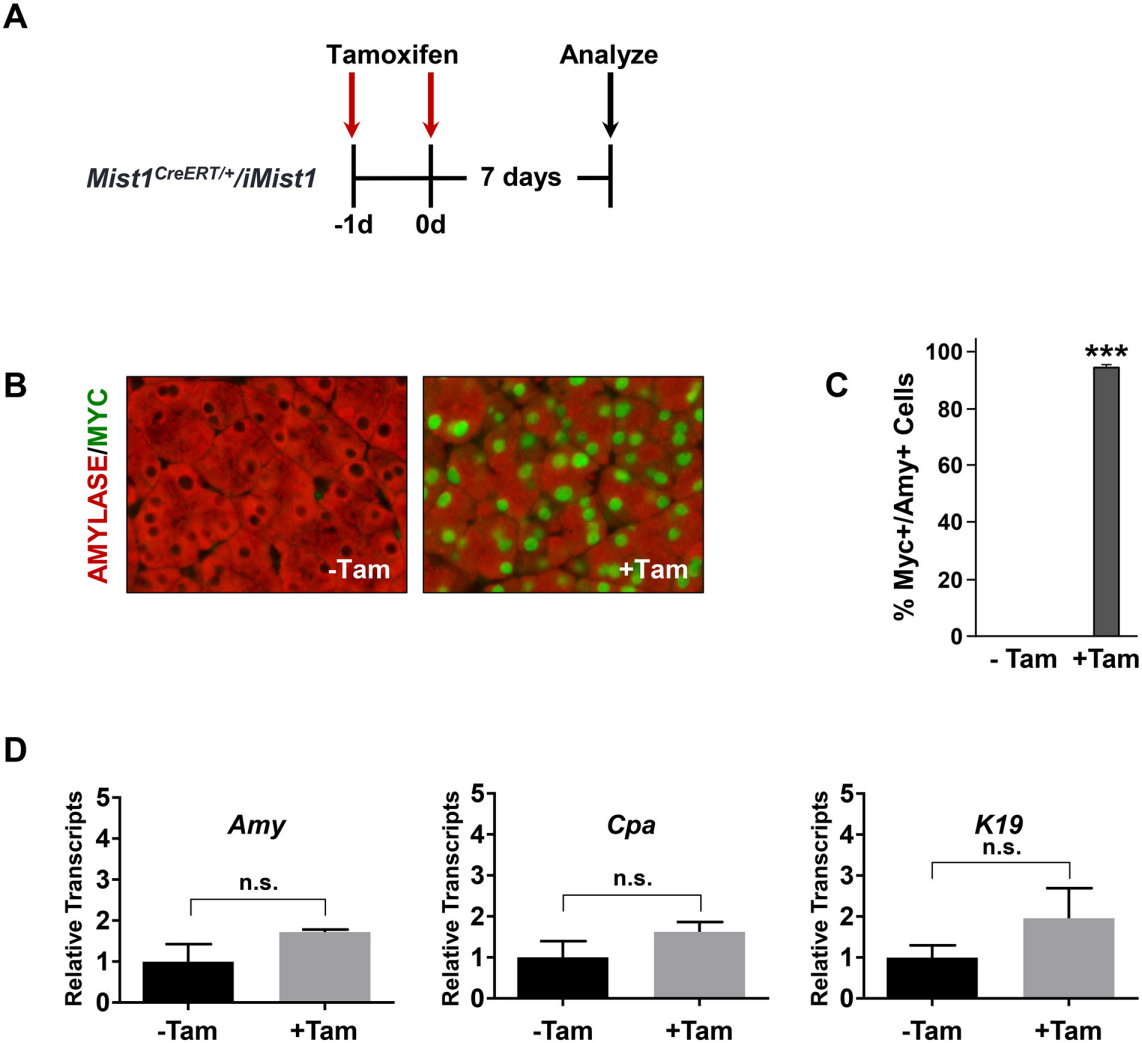
Mist1^CreERT/+^/LSL-Mist1^myc^ mice (iMist1) exhibit acinar-specific Mist1^myc^ expression upon CreER^T2^ activity. (A) Diagram outlining the time course of the study. (B) Tam treatment of *iMist1* mice leads to rapid accumulation of nuclear MIST1^myc^ protein exclusively in pancreatic acinar cells. (C) Quantification of MIST1^myc^+ acinar cells following Tam induction. (D) RT-qPCR analysis reveals no deleterious effects on general pancreas properties from *Mist1* ^*myc*^ induction. See DiRenzo *et al*. [33] for a full characterization of the *iMist1* model. ***p ≤ 0.001. n.s.—not significant.

Our studies have shown that *Mist1* expression is transiently silenced during the peak of AP damage and that *Mist1* re-expression is not required for the pancreas to recover from an AP episode. Nonetheless, given the importance of MIST1 to normal acinar cell polarity and secretory function [33, 46–49, 72, 74], we investigated if sustained MIST1 protein expression could be used to limit the initial AP damage response. Previous studies have shown that formation of ADM and PanIN lesions is significantly attenuated when *Mist1* expression is maintained in the presence of oncogenic KRAS^G12D^ [26, 39]. Therefore, we hypothesized that a similar lessening of AP damage might be achieved by maintaining MIST1 transcriptional activity. For these studies, we utilized a Cre-inducible *LSL-Mist1* ^*myc*^ (*iMist1* ^*myc*^) transgenic mouse model (S5 Fig) [33] and generated *Mist1* ^*CreERT/+*^ */iMist1* ^*myc*^ offspring. Administering Tam to *Mist1* ^*CreERT/+*^ */iMist1* ^*myc*^ mice induced *iMist1* ^*myc*^ transgene expression in 94.7% pancreatic acinar cells (Fig 5A–5C). Despite elevated levels of MIST1, *Mist1* ^*CreERT/+*^ */iMist1* ^*myc*^ mice exhibited a completely normal pancreas phenotype with no significant changes in the expression of acinar and ductal genes (Fig 5D, data not shown) [33].

**Fig 6:**
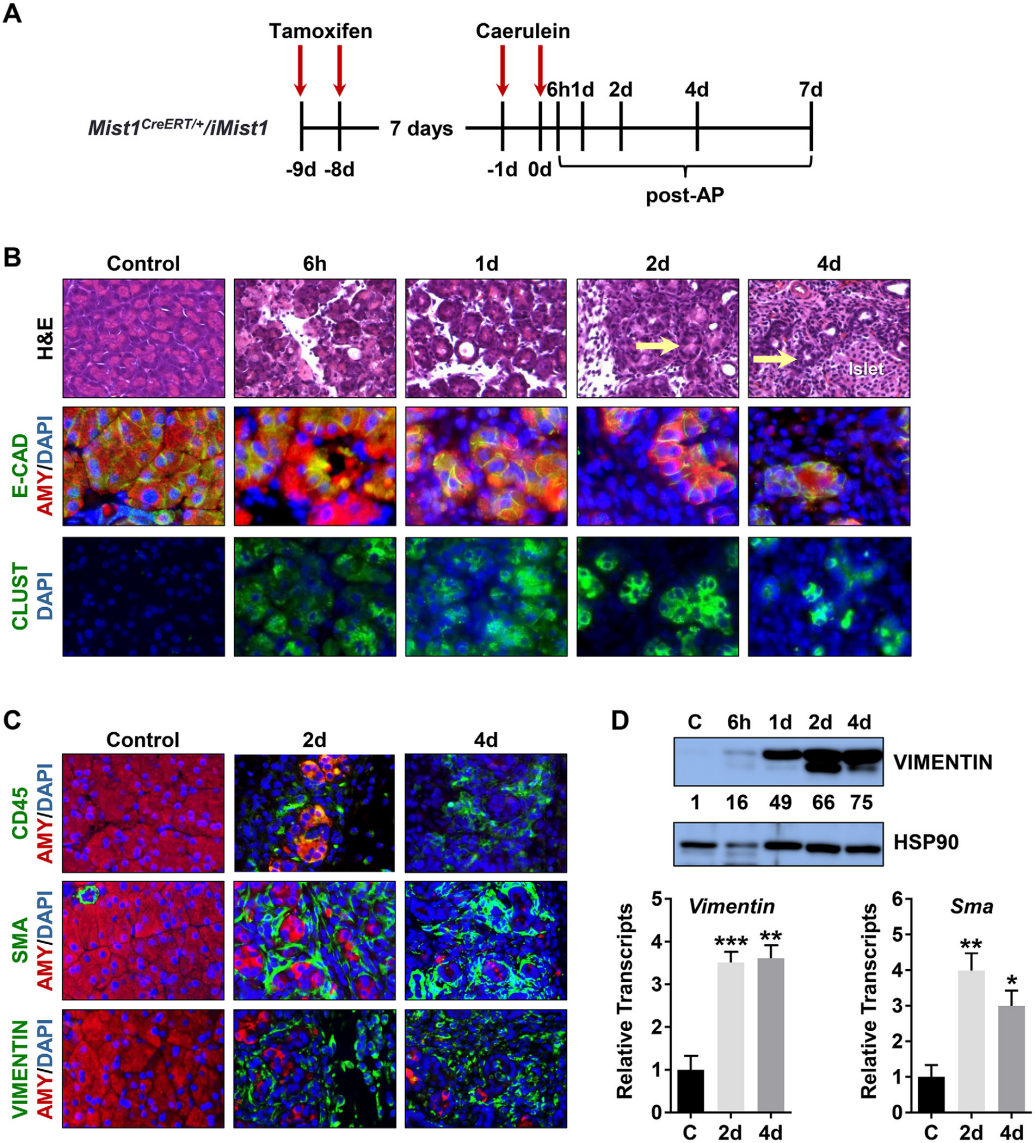
Mist1^myc^ acinar cells exhibit extensive stromal infiltrates following AP induction. (A) Time course of *iMist1* ^*myc*^ induction and AP treatment. (B) H&E and IF analysis of *iMist1* ^*myc*^ pancreata post-AP. Arrows indicate remnants of acini structures. (C) *iMist1* pancreata develop large increases in CD45+ immune infiltrates as well as VIMENTIN and SMA expressing stromal cells. (D) Immunoblot and RT-qPCR analysis of *Vimentin* and *Sma* levels in *iMist1* samples post-AP. HSP90 was used as a loading control. Relative expression levels are indicated below the VIMENTIN panel, normalized to the corresponding HSP90 signal. Note that values were rounded to the nearest whole number to accomodate lane widths. *p ≤ 0.05; **p ≤ 0.01; ***p ≤ 0.001.

**Fig 7:**
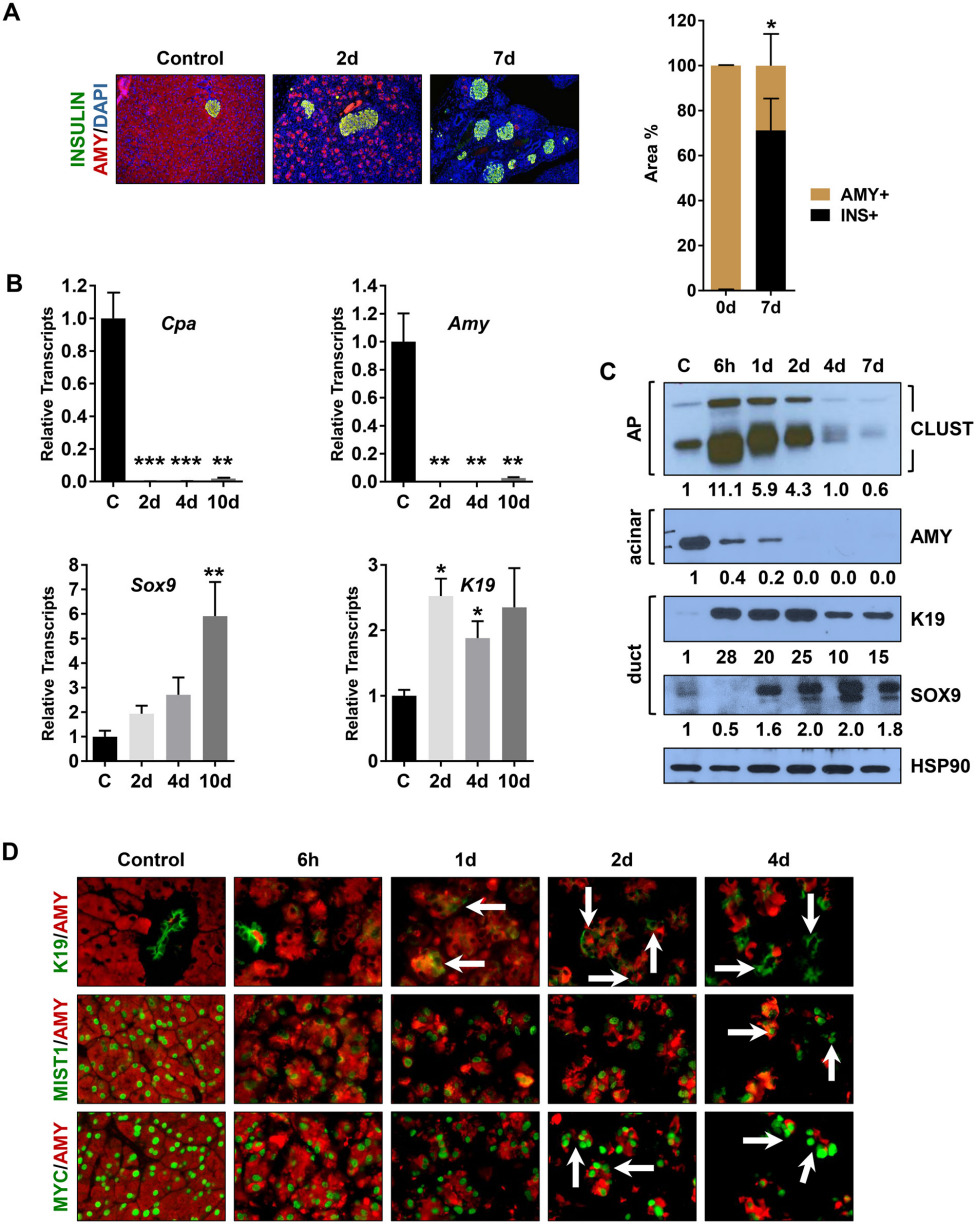
Mist1^myc^ acinar cells fail to recover from AP. (A) IF images and quantitative analysis of AMY and INS positive areas at 2d-7d post-AP. As a consequence of loosing acinar cell mass, islet tissue density increases substantially. (B) RT-qPCR analysis of ADM markers showing that *iMist1* pancreata do not recover by 10d. (C) Immunoblots revealing sustained expression of duct markers in *iMist1* samples. HSP90 was used as a loading control. Relative expression levels are indicated below each panel, normalized to the corresponding HSP90 signal. Note that K19 values were rounded to the nearest whole number to accomodate lane widths. (D) IF analysis showing the substantial loss of amylase expressing acinar cells and the persistence of K19+/AMY+ ADM lesions (arrows). Note that 4d post-AP acini structures are small with very low levels of AMY. *p ≤ 0.05; **p ≤ 0.01; ***p ≤ 0.001.

We next induced *iMist1* ^*myc*^ expression by treating *Mist1* ^*CreERT/+*^ */iMist1* ^*myc*^ mice with Tam, followed by PBS (control) or caerulein to initiate an AP phenotype (Fig 6A). Surprisingly, instead of attenuating the AP response, *Mist1* ^*CreERT/+*^ */iMist1* ^*myc*^ mice exhibited enhanced damage as early as 6h post-AP where extensive disruption of the exocrine pancreas occurred (Fig 6B and S6A Fig). By 2d-4d post-AP, the majority of acini structures were grossly altered with disorganized and distended lumens, a severe absence of eosinophilic zymogens, sustained elevated CLUSTERIN levels, and a large accumulation of infiltrating cells that included CD45+ immune cell populations (Fig 6B,6C and S6A–S6C Fig). During this period, the epithelial tissue mass was largely replaced by VIMENTIN+ and alpha-SMOOTH MUSCLE ACTIN (SMA)+ stromal cells (Fig 6C,6D and S6C Fig). The tissue also exhibited an increased islet density as the normal tissue mass that occupied space between available islets decreased, leaving the majority of the pancreas consisting of ductal, stromal and islet cells (Figs 6C and 7A). Protein immunoblots and RT-qPCR analyses revealed a typical AP damage profile with accumulation of CLUSTERIN protein, decreased expression of acinar gene products and increased expression of duct gene products over the 6h-2d post-AP period (Fig 7B–7D). However, by 7d-10d post-AP, despite reduced CLUSTERIN levels, ADM markers did not recover. Acinar genes (*Amy*, *Cpa*) remained suppressed while duct genes (*K19*, *Sox9*) continued to be expressed (Fig 7B–7D). Further analysis of these animals revealed a greatly decreased AMY+ acinar cell mass. At 2d and 4d post-AP, the vast majority of AMY+ acini co-expressed K19 in ADM structures (Fig 7D). Similarly, MIST1+ acinar cells were greatly decreased while stromal cells became more prominent within the exocrine tissue (Figs 6C, 7A, 7D and S6C Fig).

**Fig 8:**
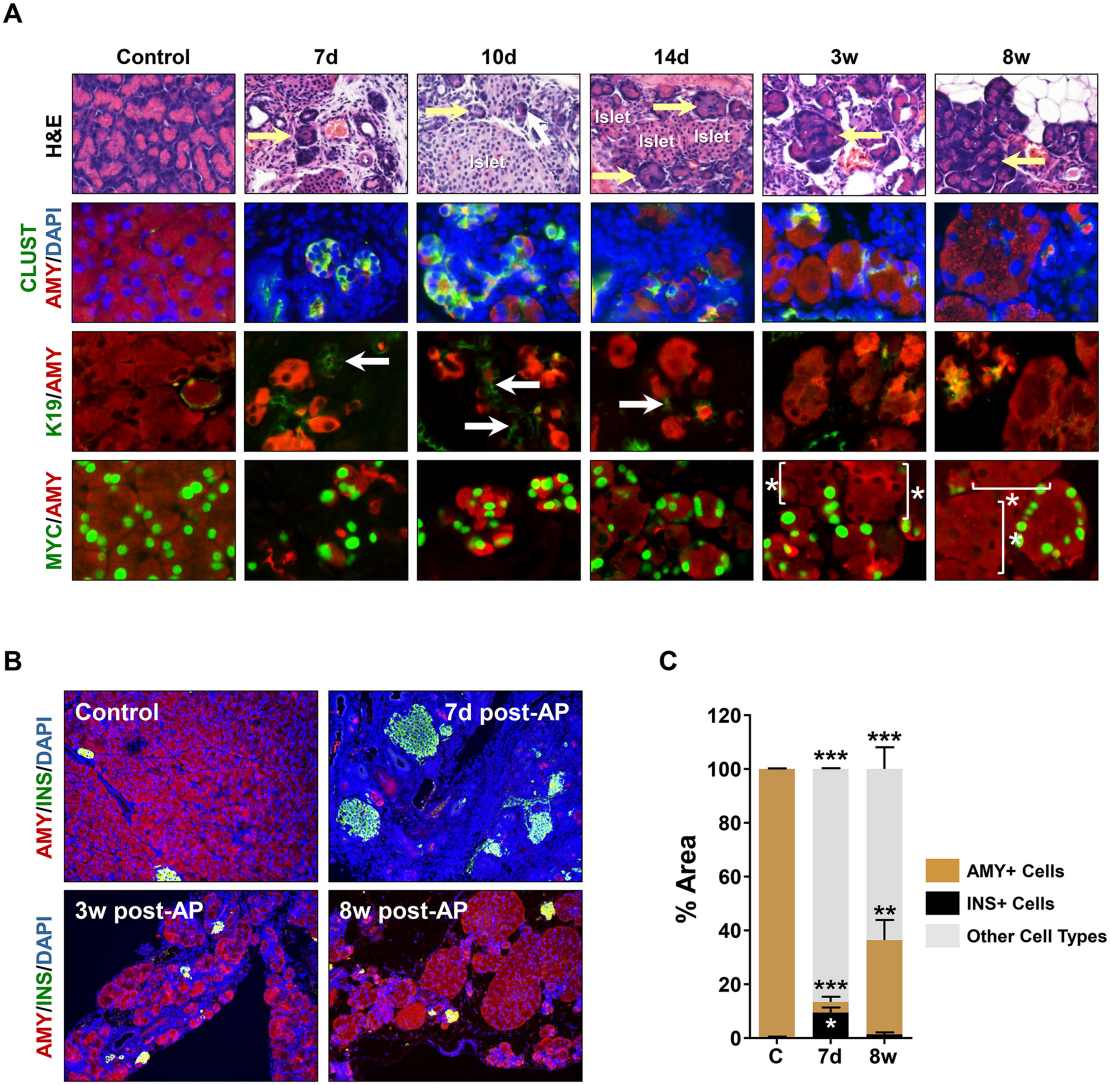
iMist1^myc^ pancreata recover from AP damage through regeneration of a minority iMist1^myc^-negative acinar cell population. (A) H&E and IF analysis of *iMist1* ^*myc*^ pancreata over the indicated post-AP time course. Arrows in the H&E images indicate acini structures that recover over the 8 week post-AP period. Arrows in the K19/AMY stained group show ADM lesions that slowly resolve by 3–8 weeks post-AP. The majority of healthy acini present at 3w-8w post-AP are MIST1^myc^ negative (brackets and asterisks). (B) IF analysis of tissue disruption and the dramatic loss of acinar cells in 7d post-AP *iMist1* ^*myc*^ pancreata followed by regeneration of Amylase+ acinar cells from 3w-8w post-AP time points. (C) Quantitative analysis of cell types associated with *iMist1* ^*myc*^ pancreata in control and 7d and 8w post-AP. **p ≤ 0.01; ***p ≤ 0.001.

**Fig 9:**
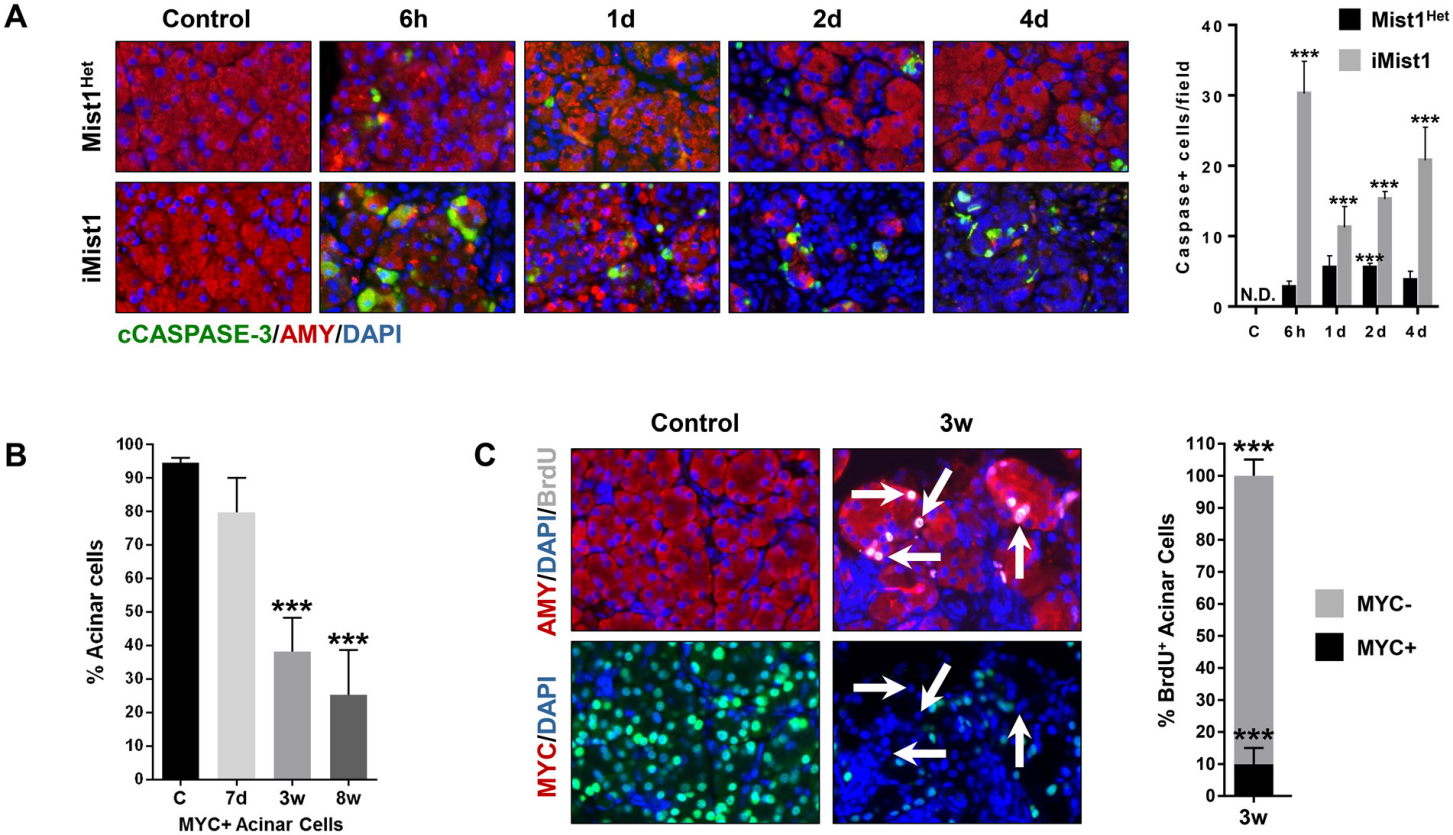
iMist1^myc^ acinar cells exhibit apoptosis followed by regeneration of iMIST1^myc^-negative cells upon AP induction. (A) Acinar cells in *iMist1* ^*myc*^ pancreata undergo extensive apoptosis as detected by cleaved CASPASE 3 staining in AMY+ cells. (B) Over time the number of MIST1^myc^+ cells is greatly decreased as the *iMist1* ^*myc*^ pancreas recovers post-AP. (C) BrdU pulse labeling reveals that regeneration of *iMist1* ^*myc*^ pancreata following AP is due to proliferation of rare acinar cells that did not activate expression of *iMist1* ^*myc*^ during the initial Tam treatment. Arrows indicate AMY+/BrdU+/MYC- cells. ***p ≤ 0.001. N.D.—not detected.

The inability of *iMist1* ^*myc*^ mice to recover from AP damage by 7d prompted us to examine animals at extended times. During 7d-10d post-AP, *iMist1* ^*myc*^ pancreata were grossly reduced in size (S7A Fig) with no evidence of normal acini structures. Instead, the tissue was composed of loose connective tissue containing VIMENTIN+ fibroblasts, CD45+ immune cells and areas of edema (S7A Fig). Within the remaining pancreas tissue, we observed small pockets of epithelial ADM structures that exhibited elevated levels of CLUSTERIN and retained co-expression of AMY and K19 (Fig 8A and S7A and S8 Figs). However, the number of AMY+ acinar cells greatly decreased over this time period with only small groupings of acinar cells remaining at 7d post-AP (Fig 8B and 8C). During this time frame there was a significant increase in cleaved CASPASE 3+/AMY+ epithelial cells, suggesting that cell death was primarily responsible for the vivid loss of acini structures (Fig 9A and S9 Fig). Over the ensuing 3–8 weeks post-AP *iMist1* ^*myc*^ pancreata underwent a significant recovery as healthy acinar tissue began to appear in the disrupted organs (S7B Fig). Areas of ADM were replaced with relatively normal acini that were AMY+ and CLUSTERIN negative (Fig 8A–8C). Interestingly, lineage-tracing revealed that the majority of the recovered acini were MIST1^myc^ negative. This was particularly evident in the later (3–8 wk post-AP) times. Quantification of these tissues showed that approximately 75% of AMY+ acinar cells did not express the iMIST1^myc^ protein (Fig 9B). The increase in AMY+/MYC- acinar cells was exclusively due to an increase in cell proliferation of the MYC- population. At 3w post-AP there was an 18.7-fold increase in BrdU-labelled cells when compared to control pancreas samples. Importantly, of the regenerating cell population >90% BrdU+ cells were MYC- (Fig 9C). At 8w post-AP pancreata also accumulated small amounts of adipose tissue that typically associated with the periphery of the organ (Fig 8A). However, the fat cells were always MYC-, demonstrating that they did not arise via an acinar cell transdifferentiation event. Taken together, these results show that sustained MIST1 protein is detrimental to AP recovery and that the *iMist1* ^*myc*^ pancreas recovers from an AP episode by relying on the small percentage of acinar cells that failed to initially activate *iMist1* ^*myc*^ expression, allowing this population to re-enter a proliferative state and repopulate the organ. We conclude that sustained *Mist1* expression does not alleviate the initial AP damage and instead is detrimental to maintaining a healthy acinar cell state under AP conditions.

## Discussion

MIST1 is a bHLH transcription factor expressed exclusively in exocrine secretory cells, including pancreatic acinar, salivary acinar and stomach zymogenic cells [48, 51, 53, 74, 75]. A number of studies have shown that MIST1 is critical to establishing intracellular apical-basal polarity, appropriate secretory vesicle formation, expansion of the ER and the ability of cells to exhibit proper regulated exocytosis of pro-digestive enzymes [33, 46–49, 56, 74]. Additionally, MIST1 is necessary for maintaining appropriate protein synthesis and processing rates when cells are under ER stress [45, 52]. In all cases, defects in MIST1 activity greatly impact the secretory function of these organs.

The importance of the MIST1 transcriptional network also has been defined in pancreatic and stomach cancer. In both systems, silencing of *Mist1* gene expression is an early event associated with metaplasia of stomach zymogenic and pancreatic acinar cells [26, 27, 58–60]. Indeed, *Mist1* silencing is one of the first events associated with *Kras*-induced pancreatic ductal adenocarcinoma (PDAC) with MIST1 negative acinar cells exhibiting early activation of EGFR signaling and downstream MAPK pathways [26, 27]. Similarly, *Mist1*-deficient acinar cells are highly sensitized to *Kras* transformation, suggesting that MIST1 plays a tumor suppressive role in the adult pancreas [26, 39]. In support of this hypothesis, sustained *Mist1* expression in the presence of oncogenic KRAS^G12D^ dramatically prevents PanIN/PDAC development [39]. A similar phenotype has been shown for the bHLH transcription factor PTF1A where deletion of *Ptf1a* also sensitizes cells to PDAC formation [40]. Thus, bHLH factors are essential for maintaining quiescent, healthy acinar cells. Indeed, altering the bHLH transcriptional network can force human PDAC tumor cells to redifferentiate into functional acinar cells [76].

Lineage tracing strategies have confirmed that mouse and human PDAC can develop from adult acinar cells upon *Kras* ^*G12D*^ and other oncogenic or tumor suppressor gene mutations [58, 77, 78]. However, despite the presence of a KRAS^G12D^ driver, most acinar cells remain refractile to transformation unless secondary stressors are placed upon the cells [28, 79]. Although loss of *Mist1* can be a secondary driver to PDAC development, there is little evidence that homozygous deletion of *Mist1* alleles occurs in PDAC patients. Instead, other pathways that result in decreased *Mist1* expression could be responsible for enhancing PDAC development. For this reason, we investigated how pancreatitis, a known risk factor for PDAC [12–14], influences *Mist1* gene expression and activity and ultimately the development of ADM lesions, the precursors to PanIN/PDAC progression. Our studies revealed that the *Mist1* locus is transiently silenced during the initial damage stage of AP. The *Mist1* gene continues to be repressed as acinar cells enter an early recovery phase during which a significant increase in cell proliferation aids the organ in regenerating. However, as this recovery continues, *Mist1* transcripts and protein return to normal levels, allowing the restored acinar tissue to resume normal secretory activity. This is in contrast to instances where AP damage is combined with KRAS^G12D^. In this setting, ADM and PanIN lesions never recover *Mist1* expression, suggesting that KRAS signaling events permanently inhibit the *Mist1* gene in a cancer setting.

Despite re-expression of *Mist1* following an AP episode, MIST1 is not necessary for acinar cells to recover from AP damage. *Mist1 cKO* acini recovered with similar kinetics as observed for *Mist1* ^*+/+*^ and *Mist1* ^*+/-*^ acinar cells, although *Mist1 cKO* cells continued to exhibit the secretory defects ascribed to *Mist1* deficient cells. The similar response of *Mist1* ^*+/-*^ and *Mist1 cKO* pancreata to AP was surprising given that previous studies have shown that *Mist1* ^*-/-*^ (*Mist1* ^*KO*^) mice display an increased sensitivity to AP with amplified damage responses and a delay in regeneration [70]. Related studies have shown that *Mist1* ^*KO*^ pancreata are highly prone to ethanol-induced pancreas damage [80], suggesting that the absence of MIST1 sensitizes acinar cells to general stress/insult events. The apparent disparity between these reports and our current results is likely due to differences in the *Mist1* model systems. In the case of *Mist1* ^*KO*^ mice, the developing and adult pancreas always lacks MIST1 protein, leading to a significantly damaged acinar cell state in post-weaned animals [33, 48]. Indeed, the enhanced stress and cell damage associated with *Mist1* ^*KO*^ pancreata highly sensitizes the organ to KRAS^G12D^-induced transformation events [26, 39]. In contrast, *Mist1 cKO* animals allow for the conditional deletion of the *Mist1* loci in adult animals so that episodes of AP occur in *Mist1* null, but otherwise healthy cells. This new model allows for the direct examination of the role of MIST1 in AP recovery in the absence of the long-term stress and injury conditions associated with germ-line *Mist1* ^*KO*^ mice. Thus, we show that deleting *Mist1* just prior to induced AP has little effect on pancreas recovery, suggesting that the increased sensitivity of *Mist1* ^*KO*^ pancreata to AP was likely due to the prior damaged status of the *Mist1* ^*KO*^ organ. In support of this hypothesis, *Mist1 cKO* mice expressed increased ADM markers over time that approached levels observed in *Mist1* ^*KO*^ animals. Interestingly, Mehmood *et al*. [73] recently showed that germ-line *Mist1* ^*KO*^ pancreata are enriched for H3K4Me3 active epigenetic marks on select genes that function within pancreatitis and PDAC pathways. Several of these genes are differentially expressed in *Mist1* ^*KO*^ animals in response to AP damage [73], demonstrating that the chronic damage and stress associated with germ-line MIST1 deficiency results in key epigenetic changes that prime cells to increased sensitivity to AP and PDAC tumor formation. Thus, we now show that the absence of MIST1 *per se* is not sufficient to produce the increased sensitivity to disease states. Rather, it is the general damage and stress conditions associated with germ-line *Mist1* ^*KO*^ acinar cells that lead to increased AP responses and PDAC development.

Given that MIST1 is critical for maintaining a healthy acinar cell state and *Mist1* gene expression is transiently silenced during AP episodes, we investigated if sustained MIST1 activity could attenuate the initial damage response. Surprisingly, acinar cells that were prevented from down-regulating *Mist1* gene expression in the early stages of AP underwent CASPASE-3 dependent apoptosis, leaving the organ grossly reduced in size with large numbers of infiltrating immune and stromal cells occupying vast areas of the pancreas. Over the initial weeks post-AP, the number of AMYLASE expressing acinar cells declined dramatically and most of the remaining cells were assembled into small acini that lacked large accumulations of zymogen granules. Sustained *iMist1* expression also kept the majority of rare surviving cells in a quiescent state, most likely due to MIST1 controlling high *p21* ^*Cip1/Waf1*^ levels [46]. This is in sharp contrast to what has been shown for PanIN/PDAC formation in *Mist1* ^*KO*^ */Kras* ^*G12D*^ pancreata [26, 39]. Here, sustained iMIST1 activity prevents PanIN development but with no signs of cell death [39]. Thus, downstream KRAS signaling pathways likely provide a survival benefit to acinar cells that retain MIST1 protein during initial ADM transitions.

**Fig 10:**
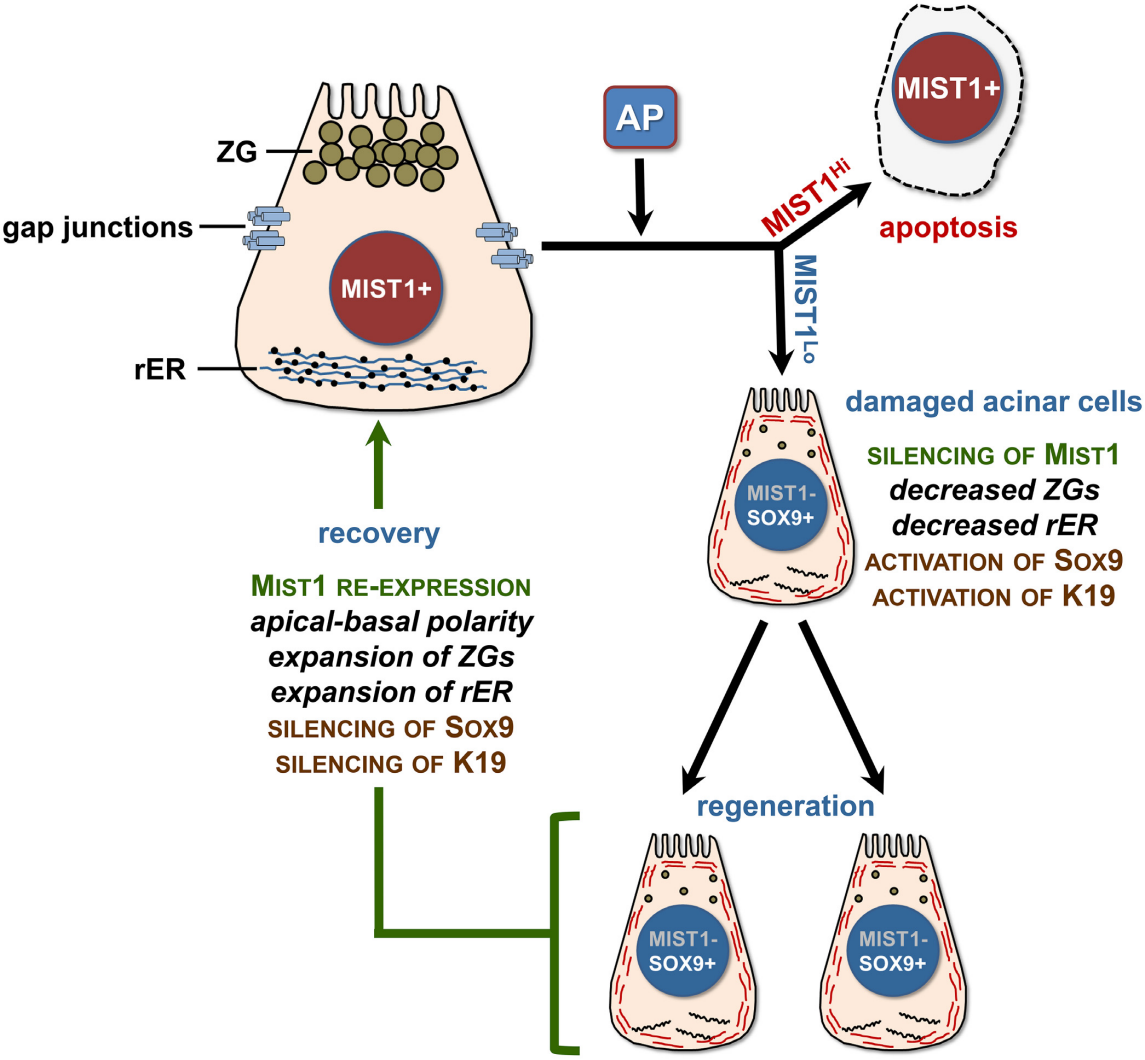
Model of Mist1 silencing and re-expression following AP recovery. For acinar cells to recover from AP damage the *Mist1* gene is required to be transiently silenced, allowing cells to reduce exocytosis function and enter a proliferative regeneration phase. Sustained *iMist1* ^*myc*^ expression during episodes of AP leads to cell death via apoptosis. ZG—zymogen granules; rER—rough endoplasmic reticulum.

Despite these widespread deficiencies, *iMist1* organs did slowly recover functional acini over time with lineage-tracing confirming that the majority of acinar cells at 8 weeks post-AP were descendants of the small percentage of cells that failed to activate expression of the *LSL-Mist1* ^*myc*^ transgene during the initial tamoxifen induction. These normal (MYC-) acinar cells that silenced *Mist1* expression during AP were able to reactive the endogenous *Mist1* gene and recover from damage. Indeed, these cells regenerated and repopulated much of the damaged pancreas in this model system. We propose that silencing *Mist1* expression is a critical event that permits acinar cells to survive an AP episode (Fig 10). Down-regulating MIST1 activity may allow cells to suppress secretory functions and *p21* ^*Cip1/Waf1*^ levels and permit a window of cell proliferation. Once established, the *Mist1* gene is then reactivated so that cells have the appropriate intracellular machinery to assemble their secretory vesicles, expand the ER, communicate via CX32-containing gap junctions, and resume efficient exocytosis functions. Thus, AP damage and recovery phases involve key transcriptional networks that control the terminal differentiation and maturation status of these specialized secretory cells. Future studies will be geared towards understanding the regulatory mechanisms that control *Mist1* expression in both AP and PDAC disease states with a long-term goal of devising strategies to modulate transcriptional networks that could alleviate clinical symptoms in patients diagnosed with pancreatitis and pancreatic cancer.

## Supporting Information

S1 Fig Quantification of AP damage in Mist1^CreERT/+^ animals. (A) Morphometric analysis of *Mist1* ^*CreERT/+*^ pancreata over the indicated times. (B) Relative CLUSTERIN, K19 and AMYLASE protein levels in IF sections from *Mist1* ^*CreERT/+*^ pancreata at the indicated times and normalized to control values. *p ≤ 0.05; **p ≤ 0.01; ***p ≤ 0.001; n.s.—not significant.
(TIF)

S2 Fig IF images of Mist1^CreERT/+^ pancreata revealing nuclear MIST1 protein exclusively in acinar cells. Islets and ducts remain MIST1 negative.
(TIF)

S3 Fig Schematic of how the Mist1^lox/+^ mice were generated through homologous recombination. LoxP sites flank the entire Mist1 coding region which is contained within exon 2.
(TIF)

S4 Fig Quantification of AP damage in Mist1^CreERT/lox^ (Mist1 cKO) animals. (A) Morphometric analysis of *Mist1 cKO* pancreata over the indicated times. (B) Relative CLUSTERIN, K19 and AMYLASE protein levels in IF sections from *Mist1 cKO* pancreata at the indicated times and normalized to control values. **p ≤ 0.01; ***p ≤ 0.001; n.s.—not significant.
(TIF)

S5 Fig Schematic of the LSL-Mist1^myc^ transgene in iMist1^myc^ mice. *Mist1* ^*CreERT/+*^ */iMist1* ^*myc*^ mice express the *iMist1* ^*myc*^ transgene exclusively in acinar cells upon Tam induction.
(TIF)

S6 Fig Quantification of AP damage in iMist1 animals 6h-4d post-AP. (A) Morphometric analysis of *iMist1* pancreata over the indicated times. (B) Relative E-CADHERIN and CLUSTERIN protein levels in IF sections from *iMist1* pancreata at the indicated times and normalized to control values. (C) Relative CD45, SMA and VIMENTIN protein levels in IF sections from *iMist1* pancreata at the indicated times and normalized to control values. *p ≤ 0.05; **p ≤ 0.01; ***p ≤ 0.001; n.s.—not significant.
(TIF)

S7 Fig H&E images of whole sections from post-AP iMist1^myc^ pancreata. (A) *Mist1* ^*CreERT/+*^ (left) and *iMist1* ^*myc*^ (right) pancreata 7d post-AP. *iMist1* ^*myc*^ pancreata contain very few Amylase+ acini structures at this time point. Inset shows a higher magnification of the boxed area. (B) *Mist1* ^*CreERT/+*^ (left) and *iMist1* ^*myc*^ (right) pancreata 8w post-AP. At this time, *iMist1* ^*myc*^ pancreata show substantial regeneration of healthy acini (arrows). Inset shows a higher magnification of the boxed area.
(TIF)

S8 Fig Quantification of AP damage in iMist1 animals 7d-8w post-AP. (A) Morphometric analysis of *iMist1* pancreata over the indicated extended times. (B) Relative CLUSTERIN, K19 and AMYLASE protein levels in IF sections from *iMist1* pancreata at the indicated times and normalized to control values. *p ≤ 0.05; **p ≤ 0.01; ***p ≤ 0.001; n.s.—not significant.
(TIF)

S1 Table Genotyping Primer Sets. (DOCX)

S2 Table Antibodies (IF and IB). (DOCX)

S3 Table RT-qPCR Primer Sets. (DOCX)
