# Supplementary material for: Silencing Mist1 Gene Expression Is Essential for Recovery from Acute Pancreatitis
Source: PLoS One. 2015 Dec 30;10(12):e0145724. doi: 10.1371/journal.pone.0145724 (PMC4696804; doi:10.1371/journal.pone.0145724)
Supplement: S1 Table — (DOCX) [file pone.0145724.s009.docx]

| **Genotype** | **Oligos** |
| --- | --- |
| *Mist1^CreER/+^* | 5'-ggttaaagcaaattgtcaagtacgg-3'; 5'- atagtaagtatgtgcgtcagcg-3'; 5'-gaagcattttccaggtatgctcag-3' |
| *LSL-Mist1^Myc^* | 5'-cgggatccttggtacctatgaag-3'; 5'-cgggatcctcagaagccatagag-3' |
| *Mist1^CreERT/lox^* | 5'- agc tgc act ggc taa gga ag-3' ; 5'-gcc ggt tct tgg tct tca ta-3';  5'-gcc ggt ttt tgg tct tca ta-3' |
