# Supplementary material for: Silencing Mist1 Gene Expression Is Essential for Recovery from Acute Pancreatitis
Source: PLoS One. 2015 Dec 30;10(12):e0145724. doi: 10.1371/journal.pone.0145724 (PMC4696804; doi:10.1371/journal.pone.0145724)
Supplement: S2 Table — (DOCX) [file pone.0145724.s010.docx]

| **Antibody** | **Species** | **Source** | **Catalog** | **Dilution (IB)** | **Dilution (IHC, IF)** |
| --- | --- | --- | --- | --- | --- |
| CLUSTERIN | goat | Santa Cruz | sc-6420 | 1:1000 | 1:200 |
| AMYLASE | goat | Santa Cruz | sc-12821 | 1:1000 |  |
| TRYPSINOGEN | mouse | Sigma | sab1400226 | 1:1000 |  |
| CYTOKERATIN-19 | rat | Dev. Studies Hybridoma Bank | troma3 | 1:1000 | 1:100 |
| SOX9 | rabbit | Millipore | ab5535 | 1:3000 | 1:4000 |
| HSP90 | rabbit | Santa Cruz | sc-7947 | 1:1000 |  |
| MIST1 (c175) | rabbit | Konieczny Lab | n/a |  | 1:500 |
| S6 | mouse | Santa Cruz | sc-74459 | 1:1000 |  |
| VIMENTIN | rabbit | Cell Signaling | 57415 | 1:1000 | 1:100 |
| MYC | mouse | Konieczny Lab | n/a |  | 1:500 |
| E-CADHERIN | rabbit | Abcam | ab53033 |  | 1:50 |
| SMA (IA4) | mouse | Santa Cruz | sc-32251 | 1:100 |  |
| CD45 | mouse | Pharminogen | 550286 | 1:50 |  |
| INSULIN | rabbit | Proteintech | 15848-1-AP |  | 1:100 |
| CONNEXIN-32 | rabbit | Abcam | ab66613 |  | 1:100 |
| BRDU | rat | Abcam | ab6326 |  | 1:100 |
| AMYLASE | rabbit | Abcam | ab21156 |  | 1:100 |
|  |  |  |  |  |  |
