# Supplementary material for: Silencing Mist1 Gene Expression Is Essential for Recovery from Acute Pancreatitis
Source: PLoS One. 2015 Dec 30;10(12):e0145724. doi: 10.1371/journal.pone.0145724 (PMC4696804; doi:10.1371/journal.pone.0145724)
Supplement: S3 Table — (DOCX) [file pone.0145724.s011.docx]

| **Gene** | **Oligos** |
| --- | --- |
| *Rplp0* | 5'-agaaactgctgcctcacatcc-3', 5'-caatggtgcctctggagatt-3 |
| *Cpa1* | 5'-ttaaaaaggcctcagacctca-3', 5'-cttcaagtgctccaactcctc-3' |
| *Amylase* | 5'-cagagacatggtgacaaggtg-3', 5'-atcgttaaagtcccaagcaga-3' |
| *K19* | 5'-cctcccgagattacaaccact-3', 5'-aggcgtgttctgtctcaaact-3' |
| *Sox9* | 5'-cacggaacagactcacatctc-3', 5'-cctctcgcttcagatcaactt-3' |
| *Mist1* | 5'-tggtggctaaagctacgtgt-3', 5'-catagctccaggctggtttt-3' |
| *Atp2c2* | 5'-ttccagactgaaaacctgagc-3', 5'-ccccttggagtggttagtaca-3' |
| *Copz2* | 5'-cttagataatgacgggcgaag-3', 5'-aagcacagacatgagcatcag-3' |
| *Rab3d* | 5'-agtgtgacctggaagacgaac-3', 5'-ccagggattcattcatcttgt-3' |
| *Rnd2* | 5'-tgtcctcaagaagtggcaag-3', 5'-tgacagggatgagtctctgc-3' |
| *Mist1 cKO* | 5'-ggctaaagctacgtgtccttg-3', 5'-tccatcttttgggagtctagg-3' |
| *Cx32* | 5'-gtgccagggaggtgtgaat-3', 5'-gataagctgcagggaccatag-3' |
| *Elastase* | 5'-gcaccgagcagtatgtgaac-3', 5'-gggagagttgttagccaggat-3' |
| *Vimentin* | 5'-tttccaagcctgacctcact-3', 5'-tccggtactcgtttgactcc-3' |
| *Sma* | 5'-tgtgctggactctggagatg-3', 5'-gcacagcttctccttgatgtc-3' |
